# Hearing impairment in systemic sclerosis patients—what do we really know?

**DOI:** 10.3389/fmed.2024.1322170

**Published:** 2024-03-18

**Authors:** Michał Sieśkiewicz, Damian Rębacz, Andrzej Sieśkiewicz

**Affiliations:** Department of Otolaryngology, Medical University of Bialystok, Bialystok, Poland

**Keywords:** hearing loss, systemic sclerosis, capillaroscopy, auditory neuropathy, cochlear impairment, cochlear diseases

## Abstract

**Background:**

Systemic sclerosis (SSc) is a disease of a very heterogeneous clinical picture and immunological profile with progression rate that varies between individuals. Although hearing deterioration is not a complaint that comes to the fore in SSc patients, as it is not life-threatening compared to many other more severe symptoms of this disease, it can significantly impair the quality of life. Medical literature concerning this problem is rather scarce.

**Materials and methods:**

In this article we systematically reviewed the medical publications concerning hearing impairment in patients with systemic sclerosis to evaluate current understanding of this complex problem. Following PRISMA guidelines a total of 19 papers were found and analysed including 11 original studies and 8 case reports.

**Results:**

Although it seems that hearing impairment in SSc patients is relatively more common than in the general population, based on the analysis of available literature, no firm conclusions regarding its frequency and pathomechanism can be drawn yet. Microangiopathy leading to damage to the sensory cells of the inner ear is suspected to be the main mechanism of hearing loss, although damage to the higher levels of the auditory pathway appears to be underestimated due to incomplete audiological diagnosis.

**Conclusion:**

Undoubtedly, the reason for the difficulty in such an evaluation are the complex and still not fully elucidated pathomechanism of SSc, the individually variable dynamics of the disease and the unique heterogeneity of symptoms. Nevertheless, further studies in larger and appropriately selected groups of patients, focused more on the dynamics of microangiopathy and not solely on clinical symptoms could provide answers to many key questions in this regard.

## 1 Introduction

Systemic sclerosis (SSc) is a chronic, multi-systemic autoimmune disease of unknown aetiology that involves connective tissue. It leads to progressive fibrosis of skin and internal organs as due to production of autoantibodies and cell-mediated autoimmunity, fibroproliferative vasculopathy of small vessels, endothelial cell damage and fibroblast dysfunction causing excessive collagen and other matrix components accumulation in skin, blood vessels, and internal organs. There are two main categories of Systemic Sclerosis: diffuse systemic sclerosis (dcSSc) and limited systemic sclerosis (lcSSc). The extent of skin involvement is used as a discriminator. Another, much less frequently described category of the disease is systemic sclerosis sine scleroderma (ssSSc) also known as noncutaneous systemic sclerosis, a subtype of scleroderma without sclerodactyly or more proximal skin involvement (1).

Microcirculatory disruption found in nailfold capillaroscopy is practically a universal symptom in patients with systemic sclerosis and is often the earliest manifestation of the disease. Destructive and proliferative vasculopathy (loss of small vessels and occlusion of arterioles and small arteries with fibro-proliferative change) observed in many internal organs such as lungs, digestive tract, kidneys and heart is a premise suggesting that inner and middle ear damage may also occur in patients with SSc leading to hearing loss secondary to the underlying disease.

SSc is a disease of a heterogeneous clinical picture with progression rate that varies between individuals. For that reason evaluation of hearing disorders in SSc patients is definitely not an easy task.

In this article we reviewed the medical publications concerning hearing impairment in patients with systemic sclerosis to find out what we really know about it.

## 2 Materials and methods

This systematic review followed the Preferred Reporting Items for Systematic Reviews and Meta-Analyses (PRISMA) guidelines. A search was conducted on 1st September 2023 across various databases including PubMed, Scopus, Web of Science, MedlinePlus, Cochrane Library, and Ingenta Connect. The aim was to explore the prevalence of hearing loss in relation to systemic sclerosis and its impact on the inner ear and auditory functions. The search strategy employed search terms such as “systemic sclerosis AND hearing loss,” “systemic sclerosis AND inner ear,” “systemic sclerosis AND auditory,” “systemic sclerosis AND cochlea” and “systemic sclerosis AND audiovestibular.” The retrieved articles were primarily in English, German, or Polish. Original research articles and case reports were included in our study, while literature reviews, letters, and editorials were excluded from consideration.

The process involved conducting searches in the aforementioned databases, retrieving articles, and excluding those that did not align with the focus of the study. The search results were organized using reference management software (Endnote 21), duplicates were removed, and the screening process was conducted independently by two authors (MS and AS). The evaluation progressed from reviewing titles to abstracts and then full-text articles in order to determine their relevance. In cases of disagreement, a third party’s input (DR) was sought.

**Figure 1:**
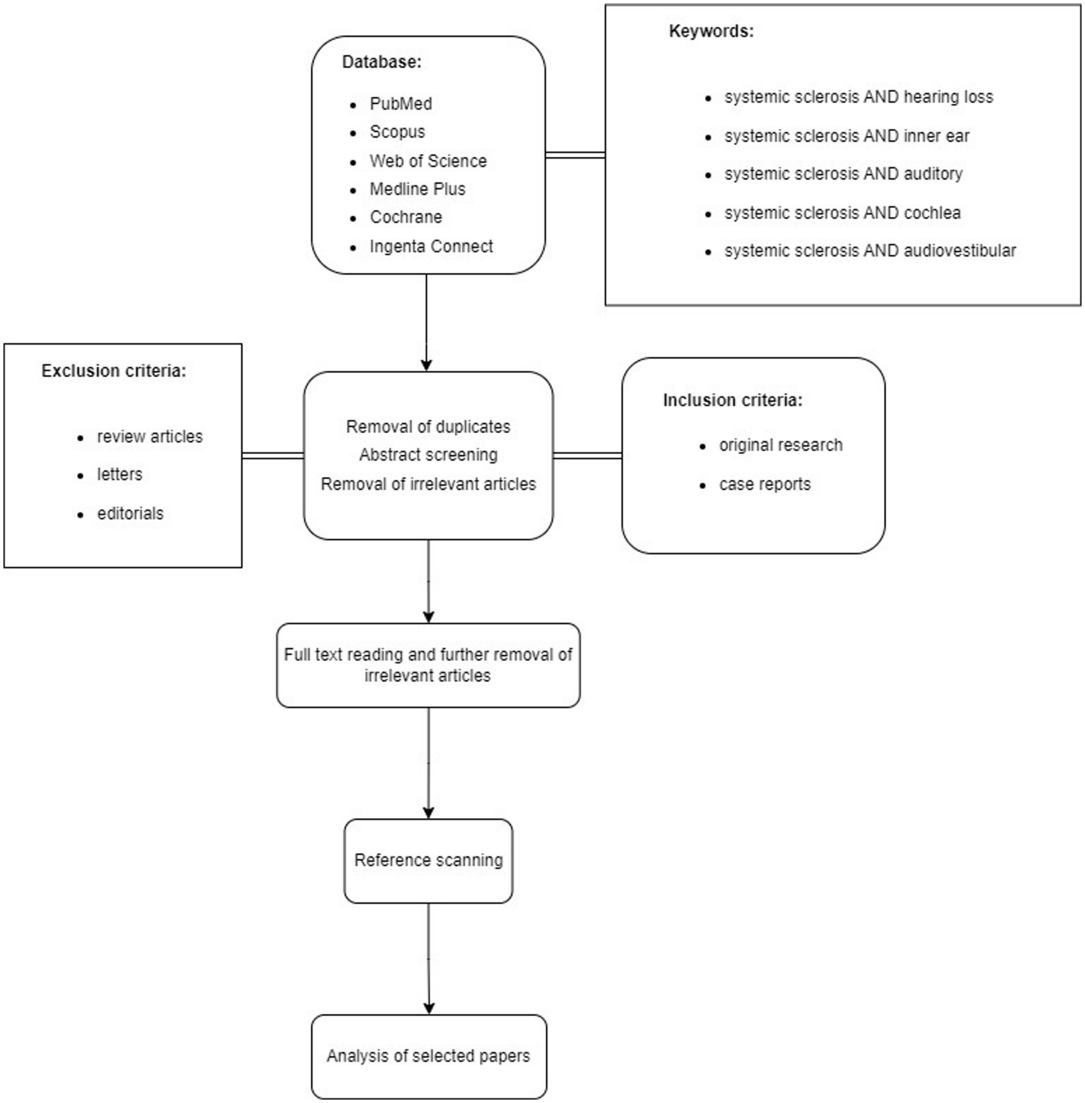
Flowchart showing the proposed approach.

It is important to note that our study specifically addressed hearing loss associated with systemic sclerosis, excluding instances linked to other vestibular or systemic conditions. Similarly, we excluded other types of hearing loss causes, such as trauma, medications, and congenital factors. Furthermore, publications not meeting specific research criteria and lacking clear methods or relying on self-report or questionnaires for hearing loss diagnosis were not considered in our review (Figure 1).

## 3 Results

**Figure 2:**
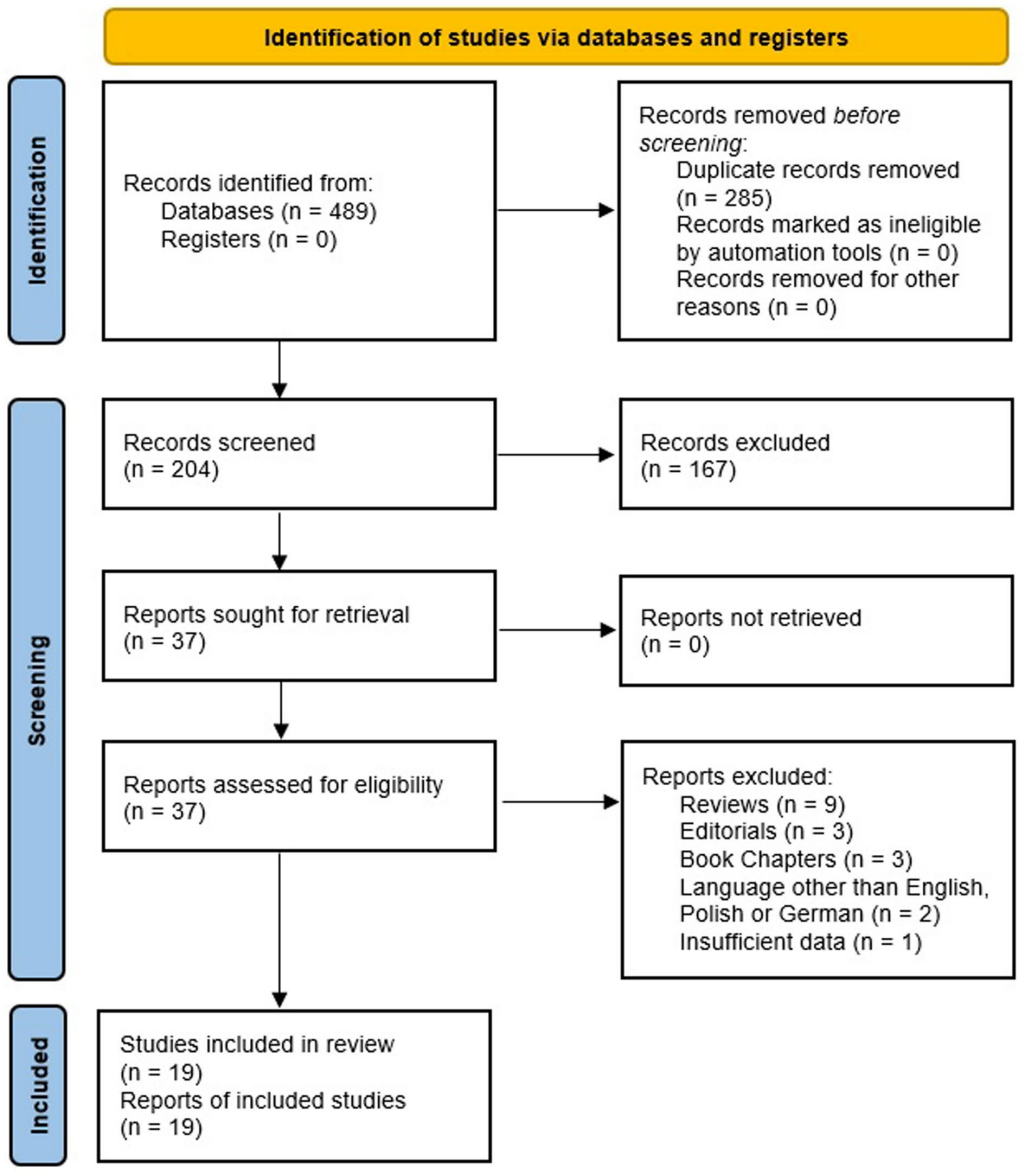
PRISMA flow diagram.

A total of 19 papers were found and analysed including 11 original studies and 8 case reports (Figure 2).

### 3.1 Group type and size, type of analysis

Sample groups of analysed original papers ranged from 8 (2) to 54 (3). Only in 3 original studies (3–5) the number of subjects exceeded 40 and in another 8 (2, 6–12) was over 20.

In 4 papers all patients with SSc, regardless the subtype, were eligible for the study (2, 5, 10, 11) in 2, data analysis of subgroups with either lcSSc (6) or dcSSc (7) was conducted and 5 authors (3, 4, 8, 9, 12) performed a comparative analysis of patients with lcSSc and dcSSc (Table 1).

**Table 1:** Studied group size, sex, presence of control group and clinical variables analysed in original studies.

	Number of patients (M/F)	Type of SSc	Sex-and age-matched control group	Duration of the disease	Involvement of internal organs	Immunological profile	Skin involvement	Nailfold capillaroscopy
Berrettini S. (1994)	37 (7/30)	lcSSc and dcSSc			X	X		
Amor-Dorado J. C. (2008)	35 (2/33)	lcSSc	X	X	X	X		
Tsirves G. (2019)	8 (2/6)	lcSSc and dcSSc		X		X		
Gheita T. (2016)	35 (0/35)	lcSSc and dcSSc	X	X	X		X	
Silva M. (2019)	50 (9/41)	lcSSc and dcSSc		X				
Monteiro T. (2011)	24 (4/20)	dcSSc	X	X	X	X	X	
Shenavandeh S. (2018)	54 (3/51)	lcSSc vs. dcSSc	X	X	X	X	X	X
Kastanioudakis I. (2001)	34 (1/33)	lcSSc vs. dcSSc	X	X		X		
Maciaszczyk K. (2010)	20 (1/19)	lcSSc vs. dcSSc	X	X	X		X	
Turan K. (2022)	47 (7/40)	lcSSc vs. dcSSc	X	X	X	X	X	X
Amor-Dorado J. C. (2023)	37 (2/35)	lcSSc and dcSSc	X	X	X	X	X	X

In analysed studies, none of the authors described any patients with systemic sclerosis sine scleroderma.

In 10 original studies the cohorts consisted of unselected, consecutive patients diagnosed with systemic sclerosis according to American College of Rheumatology/European Alliance of Associations for Rheumatology criteria, in 1 (11) only patients under 45 years of age were included. Patients with risk factors for hearing impairment other than SSc (previous audiovestibular disturbances, cranial trauma, exposure to noise, ear infection, metabolic disease, renal failure, ototoxic drug use, and familial history of hearing impairment, ear surgery, previous history of cerebrovascular complications, infections involving the inner ear etc.) were not qualified for most studies.

### 3.2 Analysed clinical features

The authors of 8 studies (3, 4, 6–9, 11, 12) compared their findings to the results of sex-and age-matched control groups. In 3 publications (2, 5, 10) no control groups were distinguished and the findings were compared to population-wide studies instead.

The relationship between hearing impairment and the duration of the disease (defined as time from the onset of symptoms) was analysed in 10 papers (2–9, 11, 12), the involvement of internal organs (lungs, kidneys, gastrointestinal tract) in 8 (3, 4, 6, 7, 9–12), immunological profile in 8 (2–4, 6–8, 10, 11) and skin involvement in accordance with Modified Rodnan skin score in 6 papers (3, 4, 7, 9, 11, 12). Nailfold capillaroscopy findings and their association with hearing loss were analysed in 3 studies (3, 4, 11) (Table 1).

### 3.3 Audiological tests

Few publications (2, 3, 5, 7, 9) addressed subjective hearing complaints. The most common symptoms reported by SSc patients were hyperacusis, tinnitus, hearing loss, ear fulness and otalgia.

The most commonly used methods of hearing evaluation were pure tone audiometry and tympanometry used in all 11 studies, stapedial reflex threshold in 10, speech audiometry—9 studies, BERA (auditory brainstem response)—3 studies, otoacoustic emissions (TEOAE)—1 study (Table 2).

**Table 2:** Audiological tests performed in original studies.

	Pure tone audiometry	Tympanometry	Stapedial reflex threshold test	Metz phenomenon	Reflex Decay test	Speech audiometry	BERA	TEOAE
Berrettini S. (1994)	X	X	X	X	X	X	X	
Amor-Dorado J. C. (2008)	X	X	X	X	X	X		
Tsirves G. (2019)	X	X	X	X				
Gheita T. (2016)	X	X	X	X		X	X	
Silva M. (2019)	X	X	X	X				
Monteiro T. (2011)	X	X	X	X		X		
Shenavandeh S. (2018)	X	X	X	X		X		
Kastanioudakis I. (2001)	X	X	X	X	X	X		
Maciaszczyk K. (2010)	X	X	X			X	X	
Turan K. (2022)	X	X				X		X
Amor-Dorado J. C. (2023)	X	X	X	X	X	X		

BERA, brainstem evoked response audiometry; TEOAE, transiently evoked otoacoustic emissions.

### 3.4 Incidence and types of hearing loss

The prevalence of hearing impairment found in audiological tests conducted in the reviewed studies ranged from 10.8% (11) to 77% (6, 12). Most often, the authors estimated it to be around 40 to 50% (5, 7, 9, 10). Diagnosed hearing loss was predominantly of sensorineural type with the site of impairment being cochlea, often bilateral, symmetrical with flat audiometric curve (4, 6, 9–12). However, some authors found a wide variety of audiogram configurations in their studies (5). In few cases (13–15) sensorineural hearing loss was related to vestibulocochlear nerve neuropathy. In a small percentage of tested patients (3, 6, 8, 10, 16, 17), usually not more than few percent, mixed or conductive hearing loss was described, though auditory tube disorders or tympanometry abnormalities were observed much more frequently (6, 8) (Table 3).

**Table 3:** Auditory findings in SSc patients.

	Patients with hearing loss (%)	Type of hearing loss
Berrettini S. (1994)	41%	Sensorineural (mostly cochlear)—71% Mixed—29% Mostly bilateral
Amor-Dorado J. C. (2008)	77%	Sensorineural, flat audiometric curve ca 50%, high frequency hearing loss ca 50% Mixed—11% 20%—abnormal tympanometry Mostly bilateral 46%—symmetrical 54% asymmetrical
Tsirves G. (2019)	75%	Sensorineural, cochlear involvement ca 50%
Gheita T. (2016)	77%	Sensorineural, bilateral, mostly cochlear
Silva M. (2019)	46%	Mostly sensorineural, variable degree, configurations of audiograms
Monteiro T. (2011)	46%	Sensorineural, cochlear involvement 54% Mostly bilateral and symmetrical
Shenavandeh S. (2018)	66.7%	Sensorineural—94.4% Mixed—2.7%, Conductive—2.7%
Kastanioudakis I. (2001)	23%	Sensorineural—86% (cochlear type) Mixed—14% 10% auditory tube dysfunction
Maciaszczyk K. (2010)	40%	Sensorineural, mostly cochlear, flat audiometric curve, high frequency hearing loss Mostly bilateral
Turan K. (2022)	28%	Mostly sensorineural - 85%, mostly cochlear Mixed type—15%
Amor-Dorado J. C. (2023)	10.8%	Sensorineural hearing loss, high frequency hearing loss—100%

### 3.5 Clinical features and their relation to hearing loss

The analysis of literature in terms of assessing the relation between hearing loss and type of SSc, involvement of internal organs or skin (modified Rodnan skin score, mRSS), duration of the disease, changes in microcirculation (capillaroscopic assessment) and presence of antibodies did not yield conclusive results. Some authors (3, 8–10) did not notice such correlations while other (4–6) reported deterioration of inner ear function depending on the duration of the disease or lung involvement. In a study by Gheita et al. (12) mRSS significantly correlated with the high frequency hearing threshold. Hearing deterioration proved to be correlated with duration of the disease in studies by Silva et al. (5) and Turan et al. (4). Whereas studies by Turan et al. (4) and Amor-Dorado et al. (6) noticed higher prevalence of hearing loss in patients with pulmonary disorders (Table 4). No correlation was found between objective hearing loss and capillaroscopy findings by Shenavandeh et al. (3) and Turan et al. (4), although more recent study by Amor-Dorado et al. (11) confirmed higher incidence of hearing loss in patients with Raynaud phenomenon secondary to systemic sclerosis.

**Table 4:** Internal organ involvement and its correlation with hearing loss.

	Internal organ involvement/clinical features	Correlation with hearing loss
Berrettini S. (1994)	Pulmonary, renal, cardiac, oesophageal involvement	No correlation
Amor-Dorado J. C. (2008)	Oesophageal dysmotility, pulmonary hypertension, pericarditis	All the patients with pulmonary hypertension were found to have hearing loss
Monteiro T. (2011)	Pulmonary fibrosis, pulmonary hypertension, oesophageal dysmotility, cardiac involvement, renal involvement	No correlation
Shenavandeh S. (2018)	Gastrointestinal involvement, interstitial lung disease, pulmonary hypertension	No correlation
Gheita T. (2016)	Pulmonary, renal, cardiac, gastrointestinal involvement (Medsger severity score, MSS)	The MSS significantly correlated with the high frequency hearing threshold
Maciaszczyk K. (2010)	Non specified	No correlation
Turan K. (2022)	Gastrointestinal involvement, interstitial lung disease, cardiac involvement	Diffusing capacity of the lungs for carbon monoxide values were significantly higher in patients with sensorineural hearing loss

Some studies evaluated possible link between immunological profile of SSc patients and hearing deterioration (2–4, 6–8, 10). However, no correlation was found in any study (Table 5).

**Table 5:** Immunological profiles of SSc patients and their correlation with hearing loss.

	Serological characteristics of SSc patients	Correlation with hearing loss
Berrettini S. (1994)	ACA, anti-Scl70	No correlation found
Amor-Dorado J. C. (2008)	ACA, anti-PM/Scl, anti-RNA-polymerase, anti-fibrillin, anti-topoisomerase I	
Tsirves G. (2019)	ANA, SSA, SSB, anti-U1RNP	
Monteiro T. (2011)	ANA, ACA, anti-Scl70	
Shenavandeh S. (2018)	ANA, ACA, anti-Scl70	
Kastanioudakis I. (2001)	ANA,SSA	
Turan K. (2022)	ANA, anti-Scl70, CENP-B, anti-PM/Scl	

### 3.6 Pharmacological treatment in analysed patients

The pharmacological treatment of patients from the studied groups was specified in 5 studies (2, 4, 7–9). Most commonly used drugs were mofetil mycophenolate, cyclophosphamide, methotrexate, azathioprine, hydroxychloroquine, steroids, PDE5-inhibitors and calcium channel blockers. Treatment regime was not described in sufficient detail to draw any conclusions regarding its influence on hearing loss.

## 4 Discussion

The review of current literature on hearing impairment in patients with SSc shows clearly that it is a problem which has not been studied in sufficient detail. Out of nearly 20 of publications we were able to find in medical databases, almost half are case reports. In part, this may be due to hearing disorders being a relatively minor inconvenience when compared to more severe symptoms of SSc associated with involvement of internal organs and microangiopathy. It is noteworthy that in analysed papers subjective hearing impairment in SSc patients was less common than the one found in audiological tests (2, 5–7, 10). In a study by Maciaszczyk et al. (9), 37% of patients with SSc and hearing loss did not report any complaints regarding hearing. Audiometric tests may reveal subclinical SNHL in SSc patients (12). In fact, in many studies, it were the vestibular disorders such as dizziness and not hearing impairment that patients reported as their main symptom (2, 5, 9). Most publications showed hearing loss to be of sensorineural type with the site of lesion located in cochlea with good speech discrimination, which indeed, in cases of minor to moderate hearing loss can be a symptom of relatively little distress.

Not only the sole amount of publications but also the number of evaluated patients remained small. Current original studies comprised sample groups ranging from 54 SSc patients in a study by Shenavandeh et al. (3) to as low as 8 in Tsirves et al. (2) research. The scarcity or rather lack of large sample groups is probably due to systemic sclerosis being a rare and progressive disorder with some manifestations drastically deteriorating patient’s general condition and increasing the risk of death. Gradual worsening of patients’ health during the course of the disease and associated difficulties in getting to the hospital for evaluation and, eventually, death of patients are also reasons for the lack of long-term observational studies. Only one study evaluated the progression of hearing impairment in patients with SSc (5). The authors of this publication managed to examine merely 12 patients out of an initial group of 27 patients after a 3 years follow-up period.

In most studies, the number of patients only slightly exceeded 20. Given the multiplicity of variables that had to be analysed due to heterogeneity of clinical manifestations, immune profiles or dynamics of SSc this amount seems to be, from a statistical standpoint, rather insufficient.

Although all of the papers reviewed reported an increased prevalence of hearing impairment in SSc patients, to identify whether hearing loss might be a disease specific symptom, it is crucial to specifically compare the frequency of observed abnormalities in the studied patients groups with the frequency of these abnormalities in the general population. However, in this case, population-wide studies of hearing do not provide good comparative material because of the specific exclusion criteria used in current literature such as congenital hearing loss, congenital anatomic abnormalities of the ear, familial history of hearing impairment, cranioencephalic trauma, meningitis, noise exposure, ototoxic drug use such as salicylates, gentamicin, and streptomycin, acoustic trauma, metabolic and immunologic diseases, etc. These are habitually omitted in general population studies. Thus most authors (3, 4, 6–9, 12) analysed their dry findings in relation to sex-and age-matched control groups.

Major differences and some inaccuracies in the results of presented studies may also be due to the chosen method of audiological diagnosis. While basic audiometric tests such as pure tone audiometry, tympanometry and stapedial reflex threshold were used almost in all original papers, more complex audiological diagnostics were performed much less frequently. Extending the diagnosis to include BERA, TEOAE but also DPOAE and high-frequency audiometry, not used in any of the previous studies, could detect more subtle, subclinical auditory pathway abnormalities that develop early in SSc patients. Maciaszczyk et al. (9) found, with ABR testing, the presence of subclinical conduction abnormalities in the cochlear nerve, previously undetected in tonal audiometry.

Although some authors suggest that the most common type of hearing loss in their study groups was sensorineural hearing loss with cochlear localisation, many patients were found to suffer damage to the extracochlear auditory pathway (5, 13–15). The mechanism of extracochlear hearing loss may involve inflammation of small vessels on the epineurium or vasa vasorum of the cochlear nerve (18). Some underestimation of extracochlear damage could be precisely related to the use of low-sensitivity audiological tests. Recent reports indicate that cochlear nerve neuropathy may be much more common in SSc patients than it was previously thought. By analogy with other peripheral nerves, it is likely to affect up to 50% of patients. However, small subclinical lesions are detected only with very accurate methods of diagnostics requiring specialised equipment (19, 20).

Conductive hearing disorders are mainly caused by vasculitis and subsequent connective tissue fibrosis within the middle ear, myopathy of the auditory tube muscles and disruption of intraossicular joints (21, 22) and inflammation similar to other autoimmune diseases (23, 24). Unfortunately, only few reports described the histopathological condition of the middle ear (10, 16). In both studies, the authors found hyperplastic mucosa with features of hyalinization and exudative fluid in the middle ear. On the other hand, Amor-Dorado et al. (6) clinically found features of myringosclerosis without ear effusion causing changes in tympanometry in their patients, and Kastanioudakis et al. (8) described cases resembling otosclerosis.

Few and scarce descriptions of middle ear lesions unsupported by histopathological or immunohistochemical studies still make us know very little about the conductive component of hearing loss in SSc. To date, the only study of the temporal bone of an SSc patient has shown clear features of vasculitis and perivascular fibrosis that caused focal atrial atrophy and malfunction of the hair cells of the organ of Corti (16). Autopsy studies of temporal bones of patients with other autoimmune diseases showed similar vascular changes in both the internal auditory artery and stria vascularis with degeneration of the organ of Corti (25).

Since the immune-inflammatory processes responsible for the development of microangiopathy could be the same for both inner organ and auditory receptor lesions, it seems reasonable to distinguish the study population according to the subtype of the disease (limited or diffuse). Although the two types of SSc differ primarily in the localisation of skin lesions, there are also some variations in inner organ involvement. In lcSSc, the manifestations appear later and usually with lesser severity, which translates into better prognosis than in dcSSc. In 7 studies (3, 4, 6–9, 12) the authors evaluated patients who were selected according to their SSc type. Unfortunately, it is not always possible to compare the results obtained in these works due to the fact that SSc is a disease with a very heterogeneous clinical picture and varied course and progression, which meant that the studied groups were often clinically heterogeneous (age of the subjects, presence of other risk factors besides SSc for hearing deterioration, presence/advancement of internal organ complications). Studies by Maciaszczyk et al. (9), Kastanioudakis et al. (8) and Gheita et al. (12) compared the frequency of hearing loss in patients with lcSSc and dcSSc but found no significant differences between both groups. On the other hand, a study by Amor-Dorado et al. (6) conducted on 35 patients with lcSSc showed abnormal hearing loss on an audiogram in 77% of the cases, in contrast to just 46% of dcSSc patients from another study by Monteiro et al. (7). The severity of audiological impairment was also higher in patients with lcSSc. This difference, however, may be a result of lcSSc cohort being at least 10 years older than the one consisting of dcSSc patients, and is probably not associated with the disease subtype.

Although the above results seem to disprove the correlation between the prevalence and extent of audiological impairment and systemic sclerosis subtype, more research is needed due to relatively small sample groups examined in currently available literature.

The same applies to the analysis of other clinical features indicative of SSc progression in the studied patients. Admittedly, most authors did not find any correlation between the involvement of internal organs (lungs, oesophagus, heart, kidneys) or skin (mRSS) and hearing loss nevertheless, the study of these correlations was often based on very small groups of patients, sometimes burdened with other risk factors for hearing loss. Moreover, some authors do not provide methods of assessing the severity of organ involvement. However, few studies managed to confirm such a relationship. In the study by Amor-Dorado et al. (6) all patients with pulmonary hypertension had hearing loss, and in the work of Turan et al. (4) diffusing capacity of the lungs for carbon monoxide values were significantly higher in the patients with SSc and sensorineural hearing loss. Another study by Gheita et al. (12) reported that Medsger severity score, mRSS and presence of teleangiectasia and peripheral neuritis significantly correlated with the high frequency hearing threshold.

SSc is a disease with varying dynamics but steady progression. Thus, it would seem that the duration of the disease must be reflected in the incidence and severity of hearing loss in the examined patients. But just as with other clinical features of SSc patients, the results are inconclusive. Studies by Silva et al. (5), Bobeica et al. (26), Turan et al. (4), Santarelli et al. (15) report progression of hearing loss over the course of the disease while other authors found no such dependency in their research (3, 8–10). Indirectly, the relationship between disease duration and hearing impairment may be suggested by the fact that patients with lcSSc have unexpectedly worse hearing test results than those with dcSSc (3, 6). LcSSc generally progresses more slowly and leads to serious organ complications over a much longer period of time. Consequently, slowly developing vasculopathy can cause gradual damage to the hearing organ. Moreover, it is in lcSSc that pulmonary hypertension is more common due to vascular changes of a slightly different pathomechanism than in dcSSc (27). Similarly, such changes may also occur in the hearing organ.

DcSSc patients with internal organ involvement usually receive immunosuppressive treatment, which may be associated with hearing improvement (16, 28). It has been proven that this type of treatment can not only stop but also reverse organ changes to some extent. Both reduction of skin lesions as assessed by mRSS (29) and improvement of lung function (30) have been described. Beneficial changes in the hearing organ during such treatment could therefore be expected.

Only few studies provide details on treatment received by the studied patients (2, 4, 7–9). Due to very small sample groups with specific form of treatment and ambiguous results, any conclusions cannot be drawn. Individual cases of hearing improvement after immunosuppressive treatment have already been described (16, 28) and Maciaszczyk et al. (9) noted no negative/toxic effects of methotrexate and cyclophosphamide in a group of patients with advanced SSc. Furthermore, a study by Tsirves et al. (2) found hearing loss not to be the result of ototoxicity of SSc treatment but rather caused by cochlear lesions of an unknown mechanism. To date, however, no clinical observational studies have been published on hearing organ changes during immunosuppressive treatment.

Nailfold capillaroscopy is an examination that evaluates the morphological changes of microcirculation and is used for early diagnosis, observation and prognosis of development of SSc (31). Many publications have confirmed the correlation of microcirculatory abnormalities, found on capillaroscopic studies, with changes in internal organs and the development of pulmonary hypertension (32–35). Unfortunately, only two studies (3, 4) evaluated the relationship between nailfold capillaroscopy findings and hearing loss in both diffuse and limited SSc. Most patients in these works had active SSc patterns visible in their capillaroscopy however, no correlation was found between the patterns or any components of capillary changes and presence or absence of objective hearing loss. The authors explain this by the large number of arteriovenous anastomoses in the nail shafts, which, unlike in the pulmonary circulation, bypass the capillaries, not contributing to capillary blood flow and have only thermoregulatory functions (3). Presence of Raynaud phenomenon in patients without systemic sclerosis does not seem to be related to hearing loss (11). On the other hand, an association between capillaroscopic findings in SSc and the presence of digital ulcers has been suggested (36, 37). Patients with digital ulcers had significantly worse hearing test results in one study (6) however, two other studies (3, 7) did not confirm the existence of this relationship.

Similarly, the correlation of patients’ immune profile (ACA, anti SCL-70 antibodies) with hearing disorders could not be demonstrated in papers investigating this problem (2–4, 6–8, 10). Regrettably, the authors of these publications were limited to only 2–3 of the most common antibodies - ANA, ACA, Scl-70 while recent studies indicate the possibility of clinical use of more than a dozen antigens related to specific disease manifestations (38). In addition, in most papers, size of the groups of patients with positive antibody tests ranged from a few to a dozen which makes statistical analysis difficult. Some of the publications cannot deny the possibility of having included patients with other connective tissue autoimmune diseases in the study group like ones with Sjogren’s syndrome with a potentially different mechanism of hearing impairment (6). Thus, it seems that further studies in appropriately selected and larger groups of patients with the use of more antibodies seem warranted, all the more so, as previous reports have shown, there is a correlation between the presence of ACA and Scl-70 antibodies and microangiopathy in patients with SSc (39).

In parallel to hearing disorders, patients with systemic sclerosis might also experience balance disorders as they were found to have a higher frequency of benign paroxysmal positional vertigo compared to controls. Additionally, they exhibited abnormalities in clinical tests of sensory interaction and balance (40). Nevertheless, this topic deserves to be discussed at greater length in a separate work.

## 5 Conclusion

Although it seems that hearing impairment in SSc patients is relatively more common than in the general population, based on the analysis of available literature, no firm conclusions regarding its frequency and pathomechanism can be drawn. Also, attempts to evaluate the correlation of all clinical manifestations of SSc, its types, patients’ immune profiles, capillaroscopy patterns or skin involvement with hearing impairment done so far have not yielded conclusive results.

Microangiopathy leading to damage to the sensory cells of the inner ear is suspected to be the main mechanism of hearing loss, although damage to the higher levels of the auditory pathway appears to be underestimated due to incomplete audiological diagnosis. Disorders of the middle ear that occur frequently in these patients are due to dysfunction of the auditory tube, disorders of the mobility of the intraossicular joints, or inflammation that is unspecified due to scanty histopathological data and intraoperative observations.

The dynamics and progression of hearing loss is difficult to estimate mostly due to the gradual deterioration of patients’ general condition and difficulty of reaching follow-up examinations and eventually the death of patients, but also because of not entirely understood influence of immunosuppressive treatment on the hearing organ.

Undoubtedly, the reason for the difficulty in such an evaluation are the complex and still not fully elucidated pathomechanism of SSc, the individually variable dynamics of the disease and the unique heterogeneity of symptoms. Nevertheless, further studies in larger and appropriately selected groups of patients, focused more on the dynamics of microangiopathy, medical treatment received by patients and not solely on clinical symptoms could provide answers to many key questions in this regard.

## Data availability statement

The original contributions presented in the study are included in the article/supplementary material, further inquiries can be directed to the corresponding author.

## Author contributions

MS: Writing – original draft, Methodology. DR: Writing – review & editing. AS: Supervision, Writing – review & editing, Methodology.
